# Capacity building among nursing and midwifery professional associations in East Africa

**DOI:** 10.1080/16549716.2022.2118173

**Published:** 2022-10-24

**Authors:** Stephen Ruhmel, Eunice Ndirangu-Mugo, Joseph Mwizerwa, Ahmed Sarki, Eunice Pallangyo

**Affiliations:** a Janssen Research & Development, LLC, Johnson & Johnson, Titusville, NJ, USA; b Aga Khan University School of Nursing and Midwifery, Nairobi, Kenya; c Aga Khan University School of Nursing and Midwifery, Kampala, Uganda; d Aga Khan University School of Nursing and Midwifery, Dar es Salaam, Tanzania

**Keywords:** Resource mobilisation, financial management, strategy, monitoring and evaluation, marketing and communications, professional development

## Abstract

**Background:**

Despite improvements in educational opportunities, policy changes, and pay raises in the nursing and midwifery professions in East Africa, poor working conditions, few professional development opportunities, and a general lack of respect for these professions predominate. These issues contribute to a low quality of care among a population with a high burden of communicable diseases. Health professional associations may help to address these challenges by providing a voice for nurses and midwives.

**Objective:**

This study evaluated the impact of a 5-year programme focused on strengthening nurses’ and midwives’ professional associations in East Africa.

**Methods:**

This study used a mixed methods design. Quantitative, cross-sectional descriptive data were captured via surveys (n = 1,266) distributed to association members. In-depth interviews (n = 65) were used to obtain qualitative data and complement the survey responses. Quantitative and qualitative data collection occurred concurrently. The results were compared to assess the impact of the programme across Uganda, Kenya, and Tanzania.

**Results:**

The programme successfully built capacity in four of five organisational capacity areas: resource mobilisation, financial management, strategy, and monitoring and evaluation. Marketing and communications, the fifth targeted area, did not show improvement. Capacity in both research and service delivery was also improved, despite the programme not providing training in these areas. In addition, collaboration among associations and their members was improved.

**Conclusion:**

These results support existing evidence on the impact of capacity building among professional nursing associations and coincide with the World Health Organization’s objectives for nursing. Future capacity building programmes should consider replicating the successful activities from this programme and investigate ways to reach more rural branches and provide tailored content. This study contributes to a small but growing body of knowledge that supports capacity building among the African health workforce.

## Background

Nurses and midwives in East Africa have benefited from substantial improvements in professional support through educational opportunities, policy changes, and pay increases over the past 15 years [1]. However, they still experience poor working conditions, few professional development opportunities, and a general lack of respect for their professions [1–4]. Research suggests that East African nurses face inadequate staffing, higher workloads, and poor quality and insufficient equipment and materials compared with nurses in high-income countries [2]. Such burdens on nurses and midwives can lead to burnout and a low quality of care delivered to patients [5]. As a result, many qualified professionals seek better working conditions and career opportunities in high-income countries, thereby further straining the healthcare system [1,6]. Current global inequalities in the availability of nursing personnel are reflected in the density of 9.1 nurses per 10,000 population in low-income countries compared with 107.7 nurses per 10,000 population in high-income economies [7]. This challenge is endemic in most sub-Saharan African countries, with 35 countries currently experiencing shortages of nurses and midwives [6]. The impact of workforce shortages is exacerbated by the high burden of disease throughout the region. In particular, the prevalence and mortality associated with communicable diseases such as HIV/AIDS remains disproportionately high in sub-Saharan Africa [8]. HIV prevalence among adults in East Africa is notably high (Kenya, 4.7%; Tanzania, 4.6%; Uganda, 5.7%) compared with West Africa (Ghana, 1.7%; Nigeria, 1.5%; Côte d’Ivoire, 2.6%) [9]. The lack of qualified nurses and midwives may result in low quality of care, which creates an additional challenge for improving the health of these nations [10,11].

Health professional associations may be able to help reverse this trend. These organisations act as a unified voice for those they represent by influencing policy through advocacy to improve service delivery, public perception, and working environments [12]. They also empower their members by providing them with both local and global information relevant to their profession [13]. Evidence suggests that building the capacity of professional associations improves their ability to provide such services, thereby improving the quality of nursing and midwifery care [12]. The World Health Organization (WHO) has also called for institutional capacity building in human resources for health. As stated in the WHO 2017 Biennium Report, capacity building can improve organisations’ governance, policy planning, monitoring and evaluation, and management [14]. This aligns with the Sustainable Development Goal Target 17, which broadly focuses on capacity building in developing countries [15].

In response to the growing shortages of nurses and midwives and increasing evidence supporting the importance of professional associations, the USA Centers for Disease Control and Prevention launched the African Health Regulatory Collaborative for Nurses and Midwives (ARC) in 2011. This 4-year initiative focused on the collaboration of national stakeholders in nursing and midwifery across 17 nations in East, Central, and Southern Africa [6]. The ARC was successful in improving national nursing regulations, increasing capacity, and improving leadership and collaboration among nursing and midwifery organisations. Specifically, internal capacity was built in leadership, management, fundraising, governance, and strategic planning [16]. This evidence focused on policy change; however, the empowerment of nurses and midwives through capacity building can be more broadly applied to improvements in other areas such as service delivery. Similar past programmes that focused on engaging nurses in low-income countries successfully improved research capacity when proper resourcing, leadership, and education were present [17]. However, few studies concerning nursing and midwifery professional associations in East Africa have been conducted.

Most existing literature focuses on the regulatory impact of capacity building among professional associations [6,16,18,19]. Other studies found that flexible classes for continuing professional development as well as mentoring and supervision from experienced midwives successfully built capacity in nursing and midwifery associations [20,21]. Opportunities exist to expand this body of evidence and explore new mechanisms for capturing outcomes [22]. To our knowledge, this is the first study to assess the impact of strengthening professional nursing and midwifery associations in the East Africa region.

### Project overview

In 2014, the Johnson & Johnson Foundation, the Aga Khan University School of Nursing and Midwifery in East Africa, and the Aga Khan Foundation East Africa partnered to launch a 5-year *Strengthening Nursing and Midwifery Associations in East Africa* programme. The programme focused on building the capacity of nine nursing and midwifery professional associations across Kenya, Uganda, and Tanzania through trainings and workshops. Targeted areas were: resource mobilisation, financial management, strategy, monitoring and evaluation, and marketing and communications. Trainings and workshops were conducted by the Aga Khan Foundation staff through in-person and online channels. Attendees primarily consisted of executive or national leadership from the participating associations, and a smaller proportion of members and branch leaders. Table 1 lists the participating organisations.

**Table 1.** Participating nursing and midwifery associations.

Kenya	Tanzania	Uganda
National Nurses Association of Kenya (NNAK)	Tanzania National Nurses Association (TANNA)	Uganda Nurses and Midwives Union (UNMU)
Midwives Association of Kenya (MAK)	Tanzania Midwives Association (TAMA)	Uganda Private Midwives Association (UPMA)
	Zanzibar Nurses Association (ZANA)	National Midwives Association of Uganda (NMAU)
		Association of Graduate Nurses and Midwives of Uganda (AGNMU)

### AIM and objectives

This study aimed to evaluate the impact of the *Strengthening* programme on professional organisations. Specifically, we evaluated the capacity built across the five targeted areas. These results were considered in the context of the trainings and workshops conducted as part of the programme (Table 2). Additionally, this study sought to inform future capacity building activities by capturing feedback and providing recommendations.

**Table 2.** Programme activities conducted with professional associations.

Activity	Description
Strategic Planning	Each association received hands-on training to create a 3–5 year strategic plan focused on defining their vision, mission, core values, main functions, governance models, value proposition, goals, and stakeholders.
Organisational Capacity Assessment (OCA)	Associations completed self-assessments using the Aga Khan Foundation OCA tool. Scores for each capacity area were used to inform the associations’ ISPs.
Institutional Strengthening Plan (ISP)	Based on the OCA, each association created an ISP with actionable steps and goals to increase internal capacity areas. The ISPs included resources, timelines, and owners for each step.
Virtual Resource Platform	This platform was developed in partnership with the Global Health Network to provide free, virtual resources for association members.
Regional Knowledge Sharing	Through annual general meetings, workshops, and various other forums, the programme allowed association members to interact with one another. Members across participating associations were enabled to share knowledge.
Development of Association Websites	Associations attended workshops to learn how to build and maintain free, simple websites to communicate with members and market to the public.
Membership Satisfaction	Surveys were administered to associations in 2016/2017 and again in 2019 to gauge the success of the associations’ actions and this programme. The results of these surveys are detailed in this study.
Media Training	The Aga Khan University Graduate School of Media and Communications led workshops to teach associations how to engage with media. This resulted in action plans focused on improving the image of nurses and midwives in each association’s country.
Marketing/Communications Planning	Association leadership attended workshops on communications and marketing to teach them how to effectively reach different stakeholders including existing members. Each association then created a communication plan.
Resource Mobilisation Training	A blended (online and in-person) learning course was provided to associations, allowing them to understand their resource needs and create a plan to obtain these resources.
Monitoring and Evaluation Training	An additional blended learning course was conducted with all associations to teach them how to create a monitoring and evaluation system. This included how to collect data, define performance indicators, and communicate results.
Financial Management Training	Online training was completed by association leadership to introduce finance. It provided basics on financial concepts and procedures, budgeting, reporting, and accounting.

## Methods

A mixed methods approach was used to obtain a broad sample of member feedback as well as specific details on programme outcomes from key leadership of the professional associations in Kenya, Tanzania, and Uganda. Quantitative, cross-sectional descriptive data were captured via a survey questionnaire distributed to association members. A qualitative design using in-depth interviews was used to complement survey data [23]. Data from mixed methods used in this study were triangulated and used to complement each other [24].

### Membership satisfaction survey

A self-administered survey questionnaire was distributed in English and Swahili to a conveniently sampled diverse set of members across all participating organisations (Appendix A). This survey was previously created by Aga Khan University for purposes of measuring membership satisfaction and reused in this study for feedback on the most valuable and desired services provided by the associations.

The sample size was calculated independently per country based on total association membership for each nation and assuming a non-response rate of 20% and design effect of 1.0. To determine the sample size, we used the estimated number of registered nurses from each country: 51,649 in Kenya, 31,618 in Tanzania, and 70,167 in Uganda [25–27]. The sample size estimates were obtained using sample size calculator for proportions of OpenEpi Version 3 software, a free and open source software for performing epidemiological statistics including sample size and power calculations [28]. The total sample size required for this study was 1,145 nurses at 95% confidence level. Of these, 382 nurses were required from Kenya, 380 from Tanzania, and 383 from Uganda.

Data were collected between May 2019 and July 2019. Data collection for both methods occurred concurrently across Uganda, Kenya, and Tanzania. Initially, members were invited to complete a digital version of the survey on SurveyMonkey, but a low response rate prompted the team to switch to pencil-and-paper surveys. Branch leadership aided in distributing surveys throughout their geographical areas.

Both paper and digital surveys were administered based on respondent preference. The survey captured demographic information, levels of satisfaction with the association, motivation for joining the association, key services expected by members, and the likelihood of membership renewal. Descriptive statistics (frequencies, percentages, means, medians, and modes) were calculated for all surveys. The association between the participants’ demographic characteristics and the technical and administrative services offered by professional associations were determined using Pearson’s Chi-square test (*X^2^*). *P-values* <0.05 were considered statistically significant. The analysis was conducted using R Framework software.

### Qualitative interviews

Individual in-depth interviews were completed with a purposive sample to provide a selection of participants who could contribute rich data for this study. The presidents, treasurers, and secretaries (or vice secretaries) of the nine associations, plus four members from each association, some of whom held leadership positions at the executive or branch level, were interviewed. Acting representatives were interviewed if individuals holding such positions were not available. Association leadership were approached to provide contact information for participants in the rural branches. The participants whose contact information was provided were invited to participate into this study and interviews were conducted with those who consented at their convenient time. Finally, the Registrars of the Nursing Councils were interviewed.

Qualified and trained research assistants conducted the interviews using an interview guide (Appendix B), with further probing questions asked to enhance understanding of the capacity building activities and outcomes. The interview guide was developed by Aga Khan University specifically for use in this study and included an exploration of programme activities in which the association had participated, the activities they felt made the largest impact, and the changes that had occurred within the association. Research assistants and researchers conducted continuous discussions during data collection to capture key issues pertinent to the data collection process. Based on these discussions, the interview guide was updated twice during the study to reorder and include additional questions on capacity and quality (e.g. ‘How has the quality of the association changed?’ and ‘Has the internal capacity of your association changed as a result of the programme?’). These changes improved the depth and quality of data in subsequent interviews.

All interviews in Kenya and Uganda were conducted in English. Nineteen of the 22 interviews in Tanzania were conducted in Swahili, which is the national language spoken fluently by most citizens. The research assistants transcribed all audio-recorded interviews verbatim, with these transcripts reviewed by the researchers. The transcripts were back translated by a professional linguist who spoke both English and Swahili. Data analysis was conducted using NVivo 12 and qualitative content analysis as described by Graneheim and Lundman guided identification of categories [29]. The analysis comprised of a focused reading and rereading of the transcripts to become familiar with the data. This was followed by a systematic analysis of the data to generate initial codes which described the content and were further reviewed to construct sub-categories and categories pertinent to study. Researchers reviewed categories against the data, and this process comprised of relocation of some codes. While the first author conducted the initial coding and engineered the analysis, each step was followed by discussion among the research team to ascertain credibility of the data. The team agreed on the final categories, some of which coincided with the target areas of the programme, and each category was defined and named to have a clear focus, scope, and purpose. Finally, the report was written to provide a compelling summary based on the data analysis. Table 3 provides an example of the data analysis process.

**Table 3.** Qualitative analysis example.

Extract from interviews	Codes	Sub-categories	Categories
*We launched a system where now a deduction of membership fees is done centrally from their salaries … As of now 4,500 members are in that system, and once we are done with enrolling all of them in this system we will be at good position*.	Member fees IT Systems Direct salary deduction	Membership Fee Management	Financial Management
*So it has increased our capacity financially on how to increase contribution fee from members and it has also improved our writing skills since we wrote proposal to some stake holders and now we have two to three stakeholders who are attracted and wish to work with us.*	Increased contributions from member fees Research grants		
*We have been very successfully on managing finances, you know funding counts more than anything, finance records keepings, we have succeeded very well, almost every branch is having finance systems in place.*	Record keeping and reporting Enabling branches with finance systems	Budgeting, Spending, and Reporting	

## Results

A total of 1,266 respondents from the three countries completed the survey. Of these, 381 respondents were from Uganda, 407 from Kenya, and 478 from Tanzania, respectively. The resulting response rate exceeded the average for pencil-and-paper surveys, which is typically below 50% [30]. The majority of survey respondents (71.4%) were female with a diploma-level education (54.9%), working in public practice at government-owned and run facilities (82.2%) (Table 4). Most of the participants had 2–5 years of work experience and a similar amount of time as association members. The mean age for participants was 37.6 ± 11 years, with the female participants significantly older than their male counterparts (39.2 ± 12 years *vs* 34.0 ± 8 years, respectively, *p-value* < 0.001). Furthermore, most of the participants (45.3%) were between 30 and 44 years of age.

**Table 4.** Demographic data of the respondents.

Characteristic	*n* (%)
**Mean age ± SD (years)**	37.6 ± 11
**Age group**	
19–29	311 (27.5)
30–44	512 (45.3)
45–54	219 (19.4)
≥55	88 (7.8)
**Gender**	
Male	360 (28.6)
Female	899 (71.4)
**Highest education attained**	
Certificate	209 (16.7)
Diploma	687 (54.9)
Bachelor’s degree	286 (22.8)
Master’s degree	64 (5.1)
PhD	6 (0.5)
**Workplace**	
Faith-based	54 (4.3)
Private	169 (13.5)
Public	1028 (82.2)
**Work experience**	
<2 years	146 (11.7)
2–5 years	313 (25.1)
5–10 years	298 (23.9)
10–20 years	237 (19.0)
>20 years	254 (20.3)
**Number of years as a member of the association**	
<2 years	266 (21.2)
2–5 years	376 (30.0)
5–10 years	284 (22.6)
10–20 years	201 (16.0)
>20 years	128 (10.2)
**Survey Responses by Association**	
Midwives Association of Kenya (MAK)	9 (0.7)
National Nurses Association of Kenya (NNAK)	398 (31.4)
Association of Graduate Nurses and Midwives of Uganda (AGNMU)	20 (1.6)
National Midwives Association of Uganda (NMAU)	20 (1.6)
Uganda Nurses and Midwives Union (UNMU)	302 (23.9)
Uganda Private Midwives Association (UPMA)	39 (3.1)
Zanzibar Nurses Association (ZANA)	19 (1.5)
Tanzania Midwives Association (TAMA)	93 (7.4)
Tanzania National Nurses Association (TANNA)	366 (28.9)

The sampling aimed to reach a variety of members across both urban and rural settings to represent the diverse membership of the participating associations. Table 4 presents the responses to the survey per country and per association. In total, we received responses from nine associations across the three countries (4 associations in Uganda, 3 from Tanzania, and 2 from Kenya). The highest responses were from associations from Tanzania (~38%), followed by associations from Kenya (32%) and Uganda (30%). We obtained more than three-quarters of the responses from the three largest associations: The National Nurses Association of Kenya (NNAK), Tanzania National Nurses Association (TANNA), and the Uganda Nurses and Midwives Union (UNMU).

From Table 5, it can be observed that about a third of participants regardless of age group, work experience, duration of membership, acknowledged the contributions of their respective associations towards education and career support, conferences and workshops, and professional certification (*p-value* <0.05). Similarly, nearly half of the participants regardless of educational attainment and the professional association agreed that the CPD programmes organised by their professional associations had an impact on their practice (*p-value* <0.05). However, regardless of educational attainment, gender, and association, most of the study participants reiterated little or no impact across the following domains: linkage to online resources, publishing opportunities, and scientific journals (*p-value* <0.05).

**Table 5.** Technical services/programmes provided by the professional associations and participants’ demographic characteristics.

Services/ Variables	Scientific journal		CPD programmes		Education and career support		Conferences and workshops		Professional certification		Linkage to online resources		Publishing opportunities	
	Yes (n, %)	No (n, %)	Yes (n, %)	No (n, %)	Yes (n, %)	No (n, %)	Yes (n, %)	No (n, %)	Yes (n, %)	No (n, %)	Yes (n, %)	No (n, %)	Yes (n, %)	No (n, %)
**Age group (years)**														
19–29	61 (19.6)	250 (80.4)	172 (55.3)	139 (44.7)	146 (46.9)	165 (53.1)	95 (30.6)	216 (69.4)	63 (20.3)	248 (79.7)	22 (7.1)	289 (92.9)	16 (5.1)	295 (94.9)
30–44	94 (18.4)	418 (81.6)	273 (53.3)	239 (46.7)	223 (43.6)	289 (56.4)	202 (39.5)	310 (60.5)	58 (11.3)	454 (88.7)	32 (6.3)	480 (93.7)	19 (3.7)	493 (96.3)
45–54	52 (23.7	167 (76.3)	121 (55.3)	98 (44.7)	86 (39.3)	133 (60.7)	93 (42.5)	126 (57.5)	32 (14.6)	187 (85.4)	9 (4.1)	210 (95.9)	16 (7.3)	203 (92.7)
≥55	14 (15.9)	74 (84.0)	55 (62.5)	33 (37.5)	27 (30.7)	61 (69.3)*	33 (37.5)	55 (62.5)*	7 (7.9)	81 (92.1)*	3 (3.4)	85 (96.6)	7 (7.9)	81 (92.1)
**Gender**														
Male	72 (20.0)	288 (80.0)	190 (52.8)	170 (47.2)	158 (43.9)	202 (56.1)	140 (38.9)	220 (61.1)	53 (14.7)	307 (85.3)	25 (6.9)	335 (93.1)	10 (2.8)	350 (97.2)
Female	169 (18.8)	730 (81.2)	490 (54.5)	409 (45.5)	362 (40.3)	537 (59.7)	348 (38.7)	551 (61.3)	113 (12.6)	786 (87.4)	46 (5.1)	853 (94.9)	50 (5.6)	849 (94.4)*
**Highest education attained**														
Certificate	34 (16.3)	175 (83.7)	112 (53.6)	97 (46.4)	81 (38.8)	128 (61.2)	73 (34.9)	136 (65.1)	41 (19.6)	168 (80.4)	12 (5.7)	197 (94.3)	9 (4.3)	200 (95.7)
Diploma	129 (18.8)	558 (81.2)	396 (57.6)	291 (42.4)	297 (43.2)	390 (56.8)	268 (39.0)	419 (61.0)	93 (13.5)	594 (86.5)	33 (4.8)	654 (95.2)	35 (5.1)	652 (94.9)
Bachelors	64 (22.4)	222 (77.6)	133 (46.5)	153 (53.5)	114 (39.9)	172 (60.1)	122 (42.7)	164 (57.3)	26 (9.1)	260 (90.9)	17 (5.9)	269 (94.1)	9 (3.2)	277 (96.9)
Masters	10 (15.6)	54 (84.4)	33 (51.6)	31 (48.4)	21 (32.8)	43 (67.2)	17 (26.6)	47 (73.4)	3 (4.7)	61 (95.3)	4 (6.3)	60 (93.7)	2 (3.1)	62 (96.9)
PhD	1 (16.7)	5 (83.3)	2 (33.3)	4 (66.7)*	3 (50.0)	3 (50.0)	2 (33.3)	4 (66.7)	0 (0.0)	6 (100.0)*	1 (16.7)	5 (83.3)	2 (33.3)	4 (66.7)*
**Workplace**														
Faith-based	13 (24.1)	41 (75.9)	27 (50.0)	27 (50.0)	19 (35.2)	35 (64.8)	23 (42.6)	31 (57.4)	6 (11.1)	48 (88.9)	1 (1.9)	53 (98.1)	4 (7.4)	50 (92.6)
Private	25 (14.8)	144 (85.2)	97 (57.4)	72 (42.6)	62 (36.7)	107 (63.3)	54 (31.9)	115 (68.1)	21 (12.4)	148 (87.6)	7 (4.1)	162 (95.9)	5 (3.0)	164 (97.0)
Public	199 (19.4)	829 (80.6)	554 (53.9)	474 (46.1)	435 (42.3)	593 (57.7)	404 (39.3)	624 (60.7)	136 (13.2)	892 (86.8)	63 (6.1)	965 (93.9)	51 (5.0)	977 (95.0)
**Work experience (years)**														
<2	27 (18.5)	119 (81.5)	80 (54.8)	66 (45.2)	66 (45.2)	80 (54.8)	38 (26.0)	108 (74.0)	33 (22.6)	113 (77.4)	12 (8.2)	134 (91.8)	7 (4.8)	139 (95.2)
2–5	56 (17.9)	257 (82.1)	173 (55.3)	140 (44.7)	148 (47.3)	165 (52.7)	118 (37.7)	195 (62.3)	43 (13.7)	270 (86.3)	21 (6.7)	292 (93.3)	15 (4.8)	298 (95.2)
5–10	48 (16.1)	250 (83.9)	150 (50.3)	148 (49.7)	108 (36.2)	190 (63.8)	109 (36.6)	189 (63.4)	35 (11.7)	263 (88.3)	14 (4.7)	284 (95.3)	12 (4.0)	286 (96.0)
10–20	54 (22.8)	183 (77.2)	129 (54.4)	108 (45.6)	98 (41.4)	139 (58.7)	105 (44.3)	132 (55.7)	28 (11.8)	209 (88.2)	14 (5.9)	223 (94.1)	9 (3.8)	228 (96.2)
>20	50 (19.7)	204 (80.3)	137 (53.9)	117 (46.1)	92 (36.2)	162 (63.8)*	120 (47.2)	134 (52.8)*	24 (9.5)	230 (90.5)*	10 (3.9)	244 (96.1)	15 (5.9)	239 (94.1)
**Duration of membership (years)**														
<2	53 (19.9)	213 (80.1)	137 (51.5)	129 (48.5)	101 (38.0)	165 (62.0)	85 (32.0)	181 (68.0)	47 (17.7)	219 (82.3)	18 (6.8)	248 (93.2)	12 (4.5)	254 (95.5)
2–5	76 (20.2)	300 (79.8)	211 (56.1)	165 (43.9)	184 (48.9)	192 (51.1)	133 (35.4)	243 (64.6)	49 (13.0)	327 (87.0)	27 (7.2)	349 (92.8)	21 (5.6)	355 (94.4)
5–10	49 (17.3)	235 (82.8)	144 (50.7)	140 (49.3)	113 (39.8)	171 (60.2)	127 (44.7)	157 (55.3)	36 (12.7)	248 (87.3)	13 (4.6)	271 (95.4)	14 (4.9)	270 (95.1)
10–20	36 (17.9)	165 (82.1)	110 (54.7)	91 (45.3)	80 (39.8)	121 (60.2)	85 (42.3)	116 (57.7)	22 (11.0)	179 (89.0)	9 (4.5)	192 (95.5)	7 (3.5)	194 (96.5)
>20	24 (18.8)	104 (81.2)	72 (56.3)	56 (43.8)	39 (30.5)	89 (69.5)*	59 (46.1)	69 (53.9)*	12 (9.4)	116 (90.6)	4 (3.1)	124 (96.9)	5 (3.9)	123 (96.1)
**Association**														
MAK	0 (0.0)	9 (100.0)	6 (66.7)	3 (33.3)	2 (22.2)	7 (77.8)	5 (55.6)	4 (44.4)	0 (0.0)	9 (100.0)	0 (0.0)	9 (100.0)	0 (0.0)	9 (100.0)
NNAK	43 (10.8)	355 (89.2)	137 (34.4)	261 (65.6)	112 (28.1)	286 (71.9)	199 (50.0)	199 (50.0)	28 (7.0)	370 (93.0)	14 (3.5)	384 (96.5)	12 (3.0)	386 (97.0)
NMAU	8 (40.0)	12 (60.0)	11 (55.0)	9 (45.0)	5 (25.0)	15 (75.0)	9 (45.0)	11 (55.0)	1 (5.0)	19 (95.0)	0 (0.0)	20 (100.0)	2 (10.0)	18 (90.0)
AGNMU	2 (10.0)	18 (90.0)	11 (55.0)	9 (45.0)	9 (45.0)	11 (55.0)	2 (10.0)	18 (90.0)	2 (10.0)	18 (90.0)	4 (20.0)	16 (80.0)	0 (0.0)	20 (100.0)
UNMU	78 (25.8)	224 (74.2)	152 (50.3)	150 (49.7)	103 (34.1)	199 (65.9)	102 (33.8)	200 (66.2)	43 (14.2)	259 (85.8)	21 (7.0)	281 (93.0)	21 (6.9)	281 (93.1)
UPMA	1 (2.6)	38 (97.4)	28 (71.8)	11 (28.2)	12 (30.8)	27 (69.2)	18 (46.2)	21 (53.8)	0 (0.0)	39 (100.0)	0 (0.0)	39 (100.0)	1 (2.6)	38 (97.4)
ZANA	2 (10.5)	17 (89.5)	15 (78.9)	4 (21.1)	14 (73.7)	5 (26.3)	1 (5.3)	18 (194.7)	0 (0.0)	19 (100.0)	0 (0.0)	19 (100.0)	0 (0.0)	19 (100.0)
TAMA	24 (25.8)	69 (74.2)	69 (74.2)	24 (25.8)	57 (61.3)	36 (38.7)	32 (34.4)	61 (65.6)	20 (21.5)	73 (78.5)	14 (15.1)	79 (84.9)	7 (7.5)	86 (92.5)
TANNA	83 (22.7)	283 (77.3)*	252 (68.9)	114 (31.1)*	206 (56.3)	160 (43.7)*	125 (34.2)	241 (65.8)*	72 (19.7)	294 (80.3)	18 (4.9)	348 (95.1)*	17 (4.6)	349 (95.4)

KEY: * = *p*-value <0.05, **CPD** = Continuing Professional Development, **MAK** = Midwives Association of Kenya, **NNAK** = National Nurses Association of Kenya, **AGNMU** = Association of Graduate Nurses and Midwives of Uganda, **NMAU** = National Midwives Association of Uganda, **UNMU** = Uganda Nurses and Midwives Union, **UPMA** = Uganda Private Midwives Association, **ZANA** = Zanzibar Nurses Association, **TAMA** = Tanzania Midwives Association, **TANNA** = Tanzania National Nurses Association.

From Table 6, the majority of the participants across all ages, educational levels, work experience, duration of membership, and associations expressed dissatisfaction with their professional associations across the following administrative domains provision of scholarships, awards and recognition, mentorships, research openings, partnerships with donors, provision of newsletters, and volunteering opportunities (*p-*value <0.05). However, concerning mentorship, about a quarter of participants working with Faith-based or private facilities expressed satisfaction with the level of mentorships they receive in their workplace (*p-*value <0.05). In addition, most members of the Zanzibar Nurses Association (ZANA) and a quarter of the members of Ugandan Nurses and Midwives Union (UNMU), Tanzania Midwives Association (TAMA), and the Tanzania National Nurses Association (TANNA) expressed satisfaction with the scholarship opportunities (*p-*value <0.05). The sample of participants from the ZANA comprises only about 1.6% of the total study population, and thus, caution needs to be applied when interpreting these results.

**Table 6.** Administrative services/programmes provided by the professional associations and participants’ demographic characteristics.

Services/ Variables	Scholarships		Mentorships		Research openings		Partnerships with donors		Awards		Newsletter		Volunteering opportunities	
	Yes (n, %)	No (n, %)	Yes (n, %)	No (n, %)	Yes (n, %)	No (n, %)	Yes (n, %)	No (n, %)	Yes (n, %)	No (n, %)	Yes (n, %)	No (n, %)	Yes (n, %)	No (n, %)
**Age group (years)**														
19–29	77 (24.8)	234 (75.2)	67 (21.5)	244 (78.5)	30 (9.7)	281 (90.3)	31 (10.0)	280 (90.0)	40 (12.9)	271 (87.1)	17 (5.5)	294 (94.5)	23 (7.4)	288 (92.6)
30–44	129 (25.2)	383 (74.8)	99 (19.3)	413 (80.7)	73 (14.3)	439 (85.7)	46 (9.0)	466 (91.0)	32 (6.3)	480 (93.7)	28 (5.5)	484 (94.5)	27 (5.3)	485 (94.7)
45–54	41 (18.7)	178 (81.3)	36 (16.4)	183 (83.6)	45 (20.6)	174 (79.4)	17 (7.8)	202 (92.2)	14 (6.4)	205 (93.6)	23 (10.5)	196 (89.5)	9 (4.1)	210 (95.9)
≥55	19 (21.6)	69 (78.4)	24 (27.3)	64 (72.7)	11 (12.5)	77 (87.5)*	11 (12.5)	77 (87.5)	7 (8.0)	81 (92.0)*	5 (5.7)	83 (94.3)	3 (3.4)	85 (96.6)
**Gender**														
Male	87 (24.2)	273 (75.8)	79 (21.9)	281 (78.1)	54 (15.0)	306 (85.0)	39 (10.8)	321 (89.2)	31 (8.6)	329 (91.4)	22 (6.1)	338 (93.9)	19 (5.3)	341 (94.7)
Female	214 (23.8)	685 (76.2)	168 (18.7)	731 (81.3)	121 (13.5)	778 (86.5)	78 (8.7)	821 (91.3)	69 (7.7)	830 (92.3)	69 (7.7)	830 (92.3)	45 (5.0)	854 (95.0)
**Highest education attained**														
Certificate	48 (23.0)	161 (77.0)	50 (23.9)	159 (76.1)	24 (11.5)	185 (88.5)	12 (5.7)	197 (94.3)	18 (8.6)	191 (91.4)	14 (6.7)	195 (93.3)	10 (4.8)	199 (95.2)
Diploma	192 (28.0)	495 (72.0)	136 (19.8)	551 (80.2)	87 (12.7)	600 (87.3)	67 (9.8)	620 (90.2)	63 (9.2)	624 (90.8)	53 (7.7)	634 (92.3)	29 (4.2)	658 (95.8)
Bachelors	49 (17.1)	237 (82.9)	47 (16.4)	239 (83.6)	45 (15.7)	241 (84.3)	23 (8.0)	263 (92.0)	17 (5.9)	269 (94.1)	20 (7.0)	266 (93.0)	18 (6.3)	268 (93.7)
Masters	9 (14.1)	55 (85.9)	13 (20.3)	51 (79.7)	16 (25.0)	48 (75.0)	9 (14.1)	55 (85.9)	1 (1.6)	63 (98.4)	5 (7.8)	59 (92.2)	6 (9.4)	58 (90.6)
PhD	0 (0.0)	6 (100.0)*	1 (16.7)	5 (83.3)	1 (16.7)	5 (83.3)*	2 (33.3)	4 (66.7)*	0 (0.0)	6 (100.0)	0 (0.0)	6 (100.0)	0 (0.0)	6 (100.0)
**Workplace**														
Faith-based	9 (16.7)	45 (83.3)	16 (29.6)	38 (70.4)	8 (14.8)	46 (85.2)	4 (7.4)	50 (93.0)	8 (14.8)	46 (85.2)	5 (9.3)	49 (90.7)	5 (9.3)	49 (90.7)
Private	32 (18.9)	137 (81.1)	43 (25.4)	126 (74.6)	18 (10.7)	151 (89.3)	17 (10.1)	152 (89.9)	10 (5.9)	159 (94.1)	9 (5.3)	160 (94.7)	10 (5.9)	159 (94.1)
Public	258 (25.1)	770 (74.9)	188 (18.3)	840 (81.7)*	148 (14.4)	880 (85.6)	96 (9.3)	932 (90.7)	82 (8.0)	946 (92.0)	79 (7.7)	949 (92.3)	49 (4.8)	979 (95.2)
**Work experience (years)**														
<2	38 (26.0)	108 (74.0)	29 (19.9)	117 (80.1)	19 (13.0)	127 (87.0)	14 (9.6)	132 (90.4)	16 (11.0)	130 (89.0)	6 (4.1)	140 (95.9)	8 (5.5)	138 (94.5)
2–5	86 (27.5)	227 (72.5)	72 (23.0)	241 (77.0)	33 (10.5)	280 (89.5)	34 (10.9)	279 (89.1)	36 (11.5)	277 (88.5)	22 (7.0)	291 (93.0)	17 (5.4)	296 (94.6)
5–10	81 (27.2)	217 (72.8)	53 (17.8)	245 (82.2)	34 (11.4)	264 (88.6)	24 (8.1)	279 (91.9)	17 (5.7)	281 (94.3)	25 (8.4)	273 (91.6)	18 (6.0)	280 (94.0)
10–20	52 (21.9)	185 (78.1)	44 (18.6)	193 (81.4)	45 (19.0)	192 (81.0)	20 (8.4)	217 (91.6)	12 (5.1)	225 (94.9)	17 (7.2)	220 (92.8)	13 (5.5)	224 (94.5)
>20	40 (15.8)	214 (84.2)*	47 (18.5)	207 (81.5)	41 (16.1)	213 (83.9)*	24 (9.5)	230 (90.5)	18 (7.1)	236 (92.9)*	22 (8.7)	232 (91.3)	8 (3.2)	246 (96.8)
**Duration of membership (years)**														
<2	67 (25.2)	199 (74.8)	52 (19.6)	214 (80.5)	41 (15.4)	225 (84.6)	29 (10.9)	237 (89.1)	32 (12.0)	234 (88.0)	16 (6.0)	250 (94.0)	13 (4.9)	253 (95.1)
2–5	96 (25.5)	280 (74.5)	83 (22.1)	293 (77.9)	40 (10.6)	336 (89.4)	37 (9.8)	339 (90.2)	31 (8.2)	345 (91.8)	23 (6.1)	353 (93.9)	22 (5.9)	354 (94.2)
5–10	71 (25.0)	213 (75.0)	58 (20.4)	226 (79.6)	45 (15.9)	239 (84.1)	27 (9.5)	257 (90.5)	19 (6.7)	265 (93.3)	22 (7.8)	262 (92.2)	18 (6.3)	266 (93.7)
10–20	41 (20.4)	160 (79.6)	31 (15.4)	170 (84.6)	32 (15.9)	169 (84.1)	14 (7.0)	187 (93.0)	10 (5.0)	191 (95.0)	18 (9.0)	183 (91.0)	10 (5.0)	191 (95.0)
>20	23 (18.0)	105 (82.0)	23 (18.0)	105 (82.0)	16 (12.5)	112 (87.5)	8 (6.3)	120 (93.7)	7 (5.5)	121 (94.5)*	12 (9.4)	116 (90.6)	2 (1.6)	126 (98.4)
**Association**														
MAK	1 (11.1)	8 (88.9)	0 (0.0)	9 (100.0)	5 (55.6)	4 (44.4)	0 (0.0)	9 (100.0)	0 (0.0)	9 (100.0)	1 (11.1)	8 (88.9)	0 (0.0)	9 (100.0)
NNAK	68 (17.1)	330 (82.9)	84 (21.1)	314 (78.9)	48 (12.1)	350 (87.9)	27 (6.8)	371 (93.2)	33 (8.3)	365 (91.7)	46 (11.6)	352 (88.4)	21 (5.3)	377 (94.7)
NMAU	2 (10.0)	18 (90.0)	3 (15.0)	17 (85.0)	5 (25.0)	15 (75.0)	5 (25.0)	15 (75.0)	1 (5.0)	19 (95.0)	0 (0.0)	20 (100.0)	0 (0.0)	20 (100.0)
AGNMU	1 (5.0)	19 (95.0)	4 (20.0)	16 (80.0)	5 (25.0)	15 (75.0)	5 (25.0)	15 (75.0)	0 (0.0)	20 (100.0)	1 (5.0)	19 (95.0)	4 (20.0)	16 (80.0)
UNMU	83 (27.5)	219 (72.5)	58 (19.2)	244 (80.8)	61 (20.2)	241 (79.8)	32 (10.6)	270 (89.4)	19 (6.3)	283 (93.7)	26 (8.6)	276 (91.4)	8 (2.7)	294 (97.4)
UPMA	5 (12.8)	34 (87.2)	19 (48.7)	20 (51.3)	3 (7.7)	36 (92.3)	4 (10.3)	35 (89.7)	1 (2.6)	38 (97.4)	0 (0.0)	39 (100.0)	2 (5.1)	37 (94.9)
ZANA	8 (42.1)	11 (57.9)	2 (10.5)	17 (89.5)	4 (21.1)	15 (78.9)	5 (26.3)	14 (73.6)	1 (5.3)	18 (94.7)	0 (0.0)	19 (100.0)	5 (26.3)	14 (73.7)
TAMA	27 (29.0)	66 (71.0)	22 (23.7)	71 (76.3)	4 (4.3)	89 (95.7)	14 (15.1)	79 (84.9)	3 (3.2)	90 (96.8)	4 (4.3)	89 (95.7)	6 (6.5)	87 (93.6)
TANNA	107 (29.2)	259 (70.8)*	57 (15.6)	309 (84.4)*	41 (11.2)	325 (88.8)*	25 (6.8)	341 (93.2)*	43 (11.8)	323 (88.2)*	15 (4.1)	351 (95.9)*	19 (5.2)	347 (94.8)*

KEY: * = *p*-value <0.05, **MAK** = Midwives Association of Kenya, **NNAK** = National Nurses Association of Kenya, **AGNMU** = Association of Graduate Nurses and Midwives of Uganda, **NMAU** = National Midwives Association of Uganda, **UNMU** = Uganda Nurses and Midwives Union, **UPMA** = Uganda Private Midwives Association, **ZANA** = Zanzibar Nurses Association, **TAMA** = Tanzania Midwives Association, **TANNA** = Tanzania National Nurses Association.

The results obtained from the quantitative data were complemented by qualitative data to provide a comprehensive picture of the programme’s impact in building capacity. In total, 65 interviews were conducted (nine presidents, nine treasurers, nine (vice) secretaries, thirty-six members, and two nursing council registrars). The Ugandan registrar was omitted as she was recently appointed and had not been involved in the programme implementation. Interviews typically lasted twenty to thirty minutes in duration. Twelve sub-categories were identified from the interview data: Membership Fee Payment; Donations and Grants; Annual General Meeting Participation; Budgeting, Spending, and Reporting; Membership Fee Management; Insurance and Loans, Creating and Updating Strategic Plans, Prioritisation of Activities; Project Management and Reporting; Research; Service Delivery; and Collaboration. This data showed that the programme built four of the five targeted organisational capacity areas: resource mobilisation, financial management, strategy, and monitoring and evaluation. Marketing and communications, although targeted by the *Strengthening* programme, was rarely cited as an area where capacity was built. These data represented the views of a diverse set of respondents across urban and rural areas, varying levels of experience and education, and a range of ages. These differing perspectives enriched the study and supported a comprehensive understanding of themes congruent across participants of varying backgrounds.

### Resource mobilisation

Participating associations commonly cited resource mobilisation as an area in which they built internal capacity through the *Strengthening* programme. This resource mobilisation universally concerned financial resources, as the organisations sought a sustainable cash flow for their operations. Organisations obtained revenue through means that were either new to that organisation or of increased focus.

### Membership fee payment

Some associations obtained membership fees from more members by ensuring more consistent payments among existing members or by growing their membership. Despite these improvements, nearly all associations still reported facing challenges in ensuring members paid their monthly/annual dues and maintaining comprehensive, up-to-date records of these payments. Organisations with large membership bases (e.g. Uganda Nurses and Midwives Union, Tanzania National Nurses Association, and National Nurses Association of Kenya) achieved substantial revenue from their members whereas smaller associations could not rely on membership dues as a significant stream. However, all associations sought to increase the number of dues-paying members for both additional cash flow and increased strength of the association.

*Resource mobilization made the most impact. To bring other [members] on board … we were seeing how we could get resources to boost the association, so it was really interesting and encouraging*. (Member, AGNMU)

### Donations and grants

Associations often sought grants and donations from partner organisations as an additional means of resource mobilisation. However, many interviewees mentioned that they now placed less priority on donations, as the *Strengthening* programme had informed them of the risks of relying on such financial streams. That is, the associations shared that they now understood that donors do not promise long-term financial sustainability and may only exist for a specific period of time; if donors served as a primary revenue stream but unexpectedly cut funding, an association may experience immediate and detrimental financial hardship. Therefore, associations had diversified their revenue streams through their resource mobilisation strategies. In addition to an increased focus on subscribing members and associated dues, associations had obtained funding by executing research and other projects within their region. Although this income may come in the form of a grant, it is not simply a donation, as research grants offer defined, finite opportunities for organisations to conduct work for a given sum of money. More established associations reported their ability to obtain such projects and grants had improved; however, all participating organisations desired to further improve their capacity in this area. In particular, the associations reported challenges in their capacity to conduct research, find research opportunities, and find partners to support their research activities. This continued to be an area of interest for resource mobilisation, and the associated capacity to conduct project work served as a limiting factor.

### Annual general meeting participation

Seven of the nine associations reported holding an annual general meeting (AGM) or conference in 2018. These events commonly drew hundreds of delegates (members and non-members) and charged an entry fee to participate. Survey respondents with different qualifications, years of association membership, and specific associations were either *‘somewhat satisfied’* or *‘satisfied’* with their AGM. ‘Conferences and workshops’ was the third-most popular response when survey respondents were questioned on the most valuable programme or service provided by the association (Table 5), further indicating the popularity of such events. Given the interest and draw of these events, associations placed more emphasis on holding a conference and charging for entry. Some associations also used this as an opportunity to enrol new members or renew existing memberships and collect membership fees. Conferences provided a means of raising money and growing associations, and therefore served as important annual activities for many associations.

Despite the impact of the resource mobilisation training delivered by the *Strengthening* programme, respondents commonly cited this as an area where they continue to require support. Although they had strong interest in conducting projects and conferences, they often lacked the partnerships and up-front capital necessary for such activities. Some also wished to support members with reduced fees as well as transportation, accommodation, and meal reimbursement for association activities, but lack the funds to do so. Despite being among the reported improvements, resource mobilisation remained a challenge across all associations.

## Financial management

### Budgeting, spending, and reporting

Treasurers and other leaders from all nine associations reported increased capacity in financial management and lobbying resulting from the *Strengthening* programme. Specifically, they reported increased capacity to budget, prioritise spending, and maintain proper reporting and financial records (including membership dues). This supported resource mobilisation efforts, as accurate records indicated active subscriptions at events. Previously, associations often prohibited non-members from attending their events; however, this presented the opportunity to obtain membership dues at the event, thereby contributing to their revenue and allowing immediate update of their records.

*As leaders we have a background of nursing which is a different profession, hence we don’t have a lot of knowledge about finances. But you find you are put in a position where you have to supervise money … so it can be challenging but since they trained us we were able to get the light on how to manage finances*. (Member with a leadership role, UNMU)

### Membership fee management

Participating associations benefited from financial management training in different ways, often depending on their maturity. Newer organisations took simple steps such as establishing their first bank account and creating payment options for members. These payment options were often cash or mobile money. More established associations improved on their record keeping, and in some cases, established more advanced means of enabling payment of membership dues. For example, TANNA partnered with the government to enable a ‘check off system’ where dues were automatically deducted from members’ paychecks. This system allowed consistent membership tracking and payment with limited ongoing effort from the association.

*We launched a system where now a deduction of membership fees is done centrally from their salaries … As of now 4,500 members are in that system, and once we are done with enrolling all of them in this system we will be at good position*. (Member with a leadership role, TANNA)

#### Insurance and Loans

As with resource mobilisation, associations reported additional challenges with financial management. While all associations had treasurers, these individuals sometimes lacked a financial background and sought more training. More generally, associations wished to strengthen their financial processes, reporting, and systems. Through the two free-text response questions in the survey (Appendix A, questions 4 and 9), members also expressed interest in more financial support, although this took a different form. These individuals wanted the associations to provide insurance and loans through Savings and Credit Cooperative Organizations, which are common in the region.

## Strategy

### Creating and updating strategic plans

Participating associations reported increased capacity to develop and execute a strategy, although some were more advanced than others. Two associations were able to create their inaugural strategic plan via the *Strengthening* programme, and immediately put it into action. Two other associations had come to the end of their 5-year plans and sought further support to develop their next iteration. One association continued to experience challenges in creating such a plan and sought additional support. The remaining associations had developed and were executing their plans. In addition, one Tanzanian association had extended their strategic planning to the branch level, trained their branches, and supported them in developing their own strategic plans.

### Prioritisation of activities

Such plans reportedly helped guide and focus the associations on their agreed priorities, which enabled action. The associations noted that these plans allowed them to set short-, medium-, and long-term priorities, thereby providing necessary direction. Many associations created more acute action plans as a result. For example, In Kenya, NNAK used their plan to prioritise working with the Ministry of Health on nursing and midwifery policy creation. They have since jointly drafted new legislation.

*We came back to the ground to help our members and to see the strategy, since we had a strategic plan, so we had to follow our strategic plan so that we could now focus on our strategy so that what we learned we put it in practice*. (Member with a leadership role, UPMA)

## Monitoring and evaluation

### Project management and reporting

Monitoring and evaluation was often shared as an area where associations had increased capacity. The associations reported they had improved their ability to track progress and report on their projects, as well as understand if they had accomplished what they set out to do. The associations also mentioned improved financial reporting, which was related to financial management capacity. Some organisations noted that they already had familiarity with monitoring and evaluation, including an officer dedicated to the function, although these associations still benefited from the training. This also helped some associations obtain new projects through stronger proposal writing and proper evaluation, meaning they were more likely to be awarded projects. Finally, self-monitoring made the organisations more aware of their own areas of improvement, and allowed them to further focus resources as appropriate. Unlike other capacity areas, monitoring and evaluation was rarely cited as an area where more support was required.

*In monitoring and evaluation, it has helped us so much to monitor our resources to evaluate our weaknesses … after this training we have made a resolution how to manage our weaknesses by evaluating ourselves*. (Member with a leadership role, UPMA)

### Additional outcomes

Three additional areas were reported to have been impacted by the programme: research, service delivery, and collaboration among other organisations that were not part of the programme. Research capacity was not part of any training; however, the associations’ focus on this area might have resulted from the resource mobilisation training wherein they considered methods of revenue generation. Medical training was not provided by the programme, but multiple respondents mentioned that their members had improved the quality of care that they provided. While this was an overall goal for the *Strengthening* programme, it was perhaps unlikely that the effect was realised in such a rapid and nuanced fashion. However, Johnson & Johnson is active in other projects in the region, including providing or sponsoring nursing and midwifery education and training. It is possible that members confused these trainings to be part of the *Strengthening* programme, thereby noting it as an impact. Finally, the programme fostered collaboration through regional knowledge sharing activities such as conferences and annual general meetings. Such events were typically hosted by a single association but invited members and non-members alike. This enabled collaboration among association members, but also among non-members, who were often representing another nursing or midwifery organisation. In turn, collaboration occurred more broadly across multiple associations, including some that were not part of the *Strengthening* programme.

## Discussion

The *Strengthening* programme succeeded in building internal capacity among participating organisations in resource mobilisation, financial management, strategy, and monitoring and evaluation. This newly-realised capacity contributed to near-term sustainability of the associations as they were able to obtain and manage numerous resources more effectively. While the programme also focused on marketing and communications, respondents did not consistently report having built capacity in these areas. Media training was discussed by some respondents as it was the last training conducted before this study; however, the associations had not had time to action this area as a result of the training. Broader marketing and communications training had been conducted a year earlier, which provided adequate time for the associations to build capacity in this area. Nonetheless, there was neither a consistently reported result from the programme nor indication as to why these activities failed to build capacity.

Research capacity was reported to have improved among the associations despite not being a targeted training area. However, during resource mobilisation training, research was discussed as a means of revenue generation, which might have increased the focus on this area. Furthermore, this increased attention to research was likely to have contributed to concerns expressed among associations that they lacked research capacity. The desire for increased research capacity was also reported in an earlier study with the Ugandan Association of Nurses and Midwives [31]. This finding was further supported by the demographics of survey participants wherein only 5.6% of individuals held a master’s or doctoral degree. As such, few members were likely to have academic experience in conducting research.

Monitoring and evaluation was unique in that it was the only capacity area where respondents did not report requiring additional support, but did report capacity improvements. This may be attributable to participants’ confusion concerning what monitoring and evaluation entailed and its related objectives. Wotela described strong interest in and challenges associated with training individuals in monitoring and evaluation in Africa, although participants often failed to internalise their learnings after attending workshops and training programmes [32]. The *Strengthening* programme provided a single blended learning course on monitoring and evaluation. Despite the data indicating that this had an impact, it is possible that some associations failed to fully grasp the breadth or depth of the activities required to conduct monitoring and evaluation. As such, the associations might not have realised this as an area in which they required additional support. Additionally, financial management had a stronger impact than anticipated. Even though most associations had an active treasurer, many individuals filling these roles did not possess the skills taught in the financial management training; therefore, the training significantly increased their capacity.

The World Health Organization (WHO) *State of the world’s nursing 2020* report highlighted the importance of collaboration among professional nursing associations and other groups (e.g. regulatory bodies and grassroots movements) [7]. The *Strengthening* programme succeeded in improving collaboration among participating associations for both members and leadership, often through these individuals attending the same events and trainings. Moreover, these professional associations exist, in part, to collaborate with lawmakers and other organisations that impact the nursing and midwifery professions. Stronger, skilled associations consequently have increased ability and credibility when approaching such organisations and may effectively influence decisions that impact the professions. The WHO report reinforced the importance of the *Strengthening* programme’s focus on regional collaboration among various organisations as well as their overall capacity.

These results broadly coincide with those of the ARC, which was also shown to build internal capacity among nursing and midwifery professional associations. Both the African Health Regulatory Collaborative for Nurses and Midwives (ARC) and the *Strengthening* programme built capacity in resource mobilisation and improved regional collaboration. However, the ARC primarily focused on nursing regulations, which was not a goal of the *Strengthening* programme [16]. The activities and results of the programme were consistent with the WHO *State of the world’s nursing 2020* report that discussed the role of capacity building, collaboration, and education in improving nursing [7]. However, little additional research exists on the impact of strengthening such professional organisations.

This study had some limitations. First, the cross-sectional and descriptive nature of our study meant that the findings may not be generalisable to African countries outside the study settings (Kenya, Tanzania, and Uganda). In addition, the convenience and purposive sampling used to recruit participants may affect generalisability of the study beyond nurses or midwives or the professional associations that participated in this study. The evaluation of the *Strengthening* programme primarily focused on qualitative data gathered from individual interviews and surveys. Such methods may introduce recall bias; however, this impact appears limited as both more- and less-reported events were distributed at various time periods throughout the 5-year programme. Moreover, these methods do not objectively measure the downstream goal of improving healthcare. Also, some interview questions were later found to be leading, yes/no questions (e.g. questions 4, 7, and 9 [Appendix B]). It is unlikely that this introduced bias as further probing revealed specific responses, clarifying what impacts were realised as a result of the programme. Further studies should consider using standard, validated instruments to measure the impact of such programmes on healthcare delivery and the health of the target patient population. Similarly, validated instruments should be used to measure other impacts in capacity, collaboration, and quality. Such data would complement qualitative data. However, we are confident that our results will inform continuous strengthening of nursing and midwifery associations in the three countries.

## Recommendations

Nursing and midwifery are an integral part of the healthcare system in East Africa and should continue to be supported and strengthened. The ARC successfully impacted national nursing regulations, increased capacity, and improved leadership and collaboration among nursing and midwifery organisations [16]. The findings from the *Strengthening* programme further suggest that support for such organisations positively impacts this cadre of healthcare workers on multiple levels. However, more research is required to holistically determine the impact professional associations have on healthcare.

Resource mobilisation, financial management, strategy, and monitoring and evaluation were the most impactful training delivered through the *Strengthening* programme and should be replicated for associations who require support in these areas. Future iterations of this or similar programmes can benefit from the lessons learned and information gathered during this evaluation. First, programmes should consider means by which they can reach branches and association members throughout the target population rather than concentrating on a major city. It may be best to repeat programme activities in different locations throughout the country, enabling members to attend with little impact to personal time or budget otherwise required to travel to cities to attend programme activities. Despite being resource-intensive, this is arguably the most effective means by which the largest number of individuals could be directly impacted by the programme. Additionally, the programme could benefit by being tailored to specific associations, as some respondents shared that programme activities were not relevant to them. Conducting a needs assessment or gap analysis at the start of the programme would allow the programme team to determine areas of most benefit for each association. Once underway, the programme team should consider regular follow-up and continued support across training activities to ensure the success of trainings and workshops, and indicate areas of need for each organisation.

## Conclusion

Nursing and midwifery in East Africa are critical components of the healthcare system but are challenged by low wages, lack of respect, and poor working conditions that lead to a ‘brain drain’ as nurses emigrate to higher income countries. By building capacity in professional associations representing this cadre, the associations may become better equipped to improve these professions. Improved ability to mobilise resources, raise funds, and manage finances supports the overall sustainability and growth of these associations. Strategic planning allows organisations to focus their resources on their top priorities, which may include influencing national policy to improve salaries and working conditions. Such efforts are further supported via rigorous monitoring and evaluation to help guide their activities and decisions. Building capacity among these and related areas within professional associations enables the represented workforce to have a cohesive voice and facilitates positive change in their environments. Ultimately, this allows nurses and midwives to provide a higher quality of care to their patients. This research adds to an existing, albeit limited, evidence-base for strengthening professional healthcare associations in Africa.

## Paper context

Nursing and midwifery professions in East Africa face considerable challenges that prevent this workforce from delivering a high quality of care. This study evaluated a 5-year capacity building programme for professional associations to build on the existing limited evidence of its impact on the professions. The results suggest capacity can be built within these associations, allowing them to better support the represented professions, which will ultimately improve service delivery.

## Appendix A. Survey

**Nursing & Midwifery Associations Membership Satisfaction Survey**

The aim of this survey is to evaluate members’ expectations and levels of satisfaction. This survey will contribute to the associations’ ability to improve service delivery, while also ensuring that the strategic objectives and activities are driven and responsive to member needs and expectations.

**Demographic Information**

Age:

____________________________________________

Gender: Male ☐ Female ☐

Professional Education: Certificate ☐ Diploma ☐ Bachelors ☐ Masters ☐ PhD ☐

Workplace: Public ☐ Private ☐ Faith based ☐

Work experience: < 2 Yrs ☐ 2 – 5 Yrs ☐5 – 10 Yrs ☐ 10 - 20 Yrs ☐ > 20 Yrs ☐

Are you a member of a nursing or midwifery association? Yes ☐ No ☐

If so, which association?

National Nurses Association of Kenya (NNAK) ☐

Midwives Association of Kenya (MAK) ☐

Tanzania National Nurses Association (TANNA) ☐

Tanzania Midwives Association (TAMA) ☐

Zanzibar Nurses Association (ZANA) ☐

Uganda Nurses and Midwives Union (UNMU) ☐

Uganda Private Midwives Association (UPMA) ☐

National Midwives Association of Uganda (NMAU) ☐

Association of Graduate Nurses and Midwives of Uganda (AGNMU) ☐

How long have you been a member of the association?

< 2 Yrs ☐ 2-5 Yrs☐ 5-10 Yrs ☐ 10-20 Yrs ☐ > 20 Yrs ☐

1.What were your reasons for joining the association? (Select all that apply)

a)Networking ☐

b)Prestige of being a member☐

c)To support the profession ☐

d)To support the association ☐

e)Annual conference ☐

f)To show I’m a professional ☐

g)Stay current on information on the profession ☐

h)My colleagues advised me ☐

i)Other (please specify)☐ __________________

2.How satisfied are you with the association’s leadership? (Select one)

a)Totally satisfied ☐

b)Somewhat satisfied ☐

c)Neutral ☐

d)Somewhat dissatisfied ☐

e)Very dissatisfied ☐

3.What programs or services do you value most in the association? (Select a maximum of 3)

a)Newsletter ☐

b)Scientific Journal ☐

c)Continuous Professional Development Programs ☐

d)Education and Career Support ☐

e)Scholarships ☐

f)Awards ☐

g)Mentorships ☐

h)Conferences and workshops ☐

i)Professional Certification ☐

j)Partnerships with donors ☐

k)Research Openings☐

l)Publishing Opportunities ☐

m)Linkage to online resources ☐

n)Volunteer opportunities ☐

4.Are there any other specific programs/services that you would like the association to offer? Please explain:

____________________________________________

____________________________________________

____________________________________________

____________________________________________

____________________________________________

____________________________________________

____________________________________________

____________________________________________

5.Did your association hold an annual conference/ annual general meeting in 2018?

A)Yes ☐

B)No ☐

6.How satisfied were you with your association’s conference/annual general meeting in 2018?

a)Totally satisfied ☐

b)Somewhat satisfied ☐

c)Neutral ☐

d)Somewhat dissatisfied ☐

e)Very dissatisfied ☐

7.What were you most impressed with at the annual conference/annual general meeting in 2018? (Select one)

a)Program ☐

b)Location ☐

c)Speakers ☐

d)Networking ☐

e)Abstract presentations ☐

f)Other (please specify) ☐

____________________________________________

8.On a scale of 1 to 10, where 1 is the least likely and 10 is the most likely, how likely would you be to recommend the association to a non-member?

1 2 3 4 5 6 7 8 9 10

9.Please let us know any other comments or suggestions you may have for the association:

____________________________________________

____________________________________________

____________________________________________

____________________________________________

____________________________________________

____________________________________________

____________________________________________

____________________________________________

Thank you for your time!

## Appendix B. Interview Guide

**Table:**

Association Name	
Interviewee Position	
Interviewer Name	
Date	

Introduction

Hello my name is XXX from the Aga Khan University’s School of Nursing and Midwifery. This interview is being conducted as part of an assessment of the Strengthening Nursing and Midwifery Associations in East Africa programme, which was carried out in partnership with the Aga Khan University’s School of Nursing and Midwifery (AKU-SONAM), the Aga Khan Foundation (AKF) and Johnson & Johnson (J&J). The research will analyse the extent to which the programme has achieved its objectives. The research will also identify lessons learned that will shape future programming in the region and beyond. Thank you for agreeing to participate. We are very interested to hear your valuable opinion on the Strengthening Nursing and Midwifery Associations in East Africa programme.

Questions

First, I would like to discuss the **benefits and challenges** of participating in the Strengthening Nursing and Midwifery Associations programme

1. What activities did your association participate in as part of the Strengthening Nursing and Midwifery Associations in East Africa programme?
2. Which activities made the most significant impact on your association and why?
3. Did you face any challenges with the programme? Please explain.
4. Have changes taken place within your association as a result of the programme? Please explain.
5. In your opinion, to what extent has the programme impacted the image of the nursing and midwifery professions in East Africa?
6. In your experience, to what extent has the programme influenced/shaped the services offered by your association?
7. Has regional collaboration among nursing and midwifery associations changed as part of this initiative? If yes, please share examples.
8. How has the quality of the association changed? This could include the quality of services delivered, quality of training provided, etc. Please share examples.
9. Has the internal capacity of your association changed as a result of the program? If so, what has and has not changed?

Second, I would like to ask you for any **recommendations** you have for future programming

1. Are there are areas of support you feel nursing and midwifery associations still need? Please explain.
2. If this initiative was to be replicated (either in East Africa or another geography) what recommendations would you make? Which aspects of the initiative should be changed? Which aspects of the initiative should remain?
